# Widespread White Matter Microstructure Alterations Based on Diffusion Tensor Imaging and Diffusion Kurtosis Imaging in Patients With Pontine Infarction

**DOI:** 10.3389/fnagi.2021.758236

**Published:** 2021-12-15

**Authors:** Ying Wei, Caihong Wang, Jingchun Liu, Peifang Miao, Sen Wei, Yingying Wang, Luobing Wu, Boyan Xu, Shaoqiang Han, Yarui Wei, Kaiyu Wang, Jingliang Cheng

**Affiliations:** ^1^Department of Magnetic Resonance Imaging (MRI), The First Affiliated Hospital of Zhengzhou University, Zhengzhou, China; ^2^Department of Neuro-Interventional Radiology, The First Affiliated Hospital of Zhengzhou University, Zhengzhou, China; ^3^Tianjin Key Laboratory of Functional Imaging, Department of Radiology, Tianjin, China; ^4^The First Affiliated Hospital of Henan University of Science and Technology, Luoyang, China; ^5^Beijing Intelligent Brain Cloud, Inc., Beijing, China; ^6^GE Healthcare MR Research, Beijing, China

**Keywords:** pontine infarction, white matter tracts, diffusion tensor imaging, diffusion kurtosis imaging, behavioral function

## Abstract

Neurological deficits after stroke are closely related to white matter microstructure damage. However, secondary changes in white matter microstructure after pontine infarction (PI) in the whole brain remain unclear. This study aimed to investigate the correlation of diffusion kurtosis imaging (DKI)-derived diffusion and kurtosis parameters of abnormal white matter tracts with behavioral function in patients with chronic PI. Overall, 60 patients with unilateral chronic PI (33 patients with left PI and 27 patients with right PI) and 30 normal subjects were recruited and underwent DKI scans. Diffusion parameters derived from diffusion tensor imaging (DTI) and DKI and kurtosis parameters derived from DKI were obtained. Between-group differences in multiple parameters were analyzed to assess the changes in abnormal white matter microstructure. Moreover, we also calculated the sensitivities of different diffusion and kurtosis parameters of DTI and DKI for identifying abnormal white matter tracts. Correlations between the DKI-derived parameters in secondary microstructure changes and behavioral scores in the PI were analyzed. Compared with the NC group, both left PI and right PI groups showed more extensive perilesional and remote white matter microstructure changes. The DKI-derived diffusion parameters showed higher sensitivities than did the DTI-derived parameters. Further, DKI-derived diffusion and kurtosis parameters in abnormal white matter regions were correlated with impaired motor and cognitive function in patients with PI. In conclusion, PI could lead to extensive white matter tracts impairment in perilesional and remote regions. Further, the diffusion and kurtosis parameters could be complementary for identifying comprehensive tissue microstructural damage after PI.

## Introduction

Pons is the most common site of the posterior circulation stroke, and pontine infarction (PI) accounts for approximately 7% of all ischemic strokes (Huang et al., 2019). Some patients with chronic PI exhibit behavioral dysfunctions, such as motor and cognition impairment (Maeshima et al., 2012). However, the underlying mechanism is presently unknown. Previous studies on brain injuries after PI primarily emphasized the pathological alterations of neuronal cells in gray matter and revealed extensive structural and functional changes in gray matter regions, some of which are related to behavioral impairment and recovery (Jiang et al., 2019; Wei et al., 2020a,b). White matter acts as a relay station for the central nervous system and is responsible for the information exchange and communication between different gray matter areas. However, there have been relatively few studies regarding the effects of PI on white matter microstructure.

Diffusion tensor imaging (DTI) has been used to explore secondary white matter tissue changes after PI. Progressive anterograde and retrograde degeneration in pyramidal tracts on DTI is an important neural mechanism underlying impaired motor function after PI (Liang et al., 2008). However, DTI relies on the Gaussian distribution of the movement of water molecules to quantify microstructural changes. This maybe inaccurate because water molecules tend to follow non-Gaussian diffusion due to complex microenvironments, such as cell membranes, intracellular and extracellular spaces in the brain tissue (Choudhri et al., 2013). As an extension of DTI, diffusion kurtosis imaging (DKI) can quantify the non-Gaussian distribution of water molecules and thus provide more valuable information for evaluating white matter microstructure (Jensen et al., 2005; Hui et al., 2008). Although DKI has been preliminarily applied in stroke assessment (Jensen et al., 2011; Chen et al., 2018; Shen et al., 2019), there is currently limited information regarding secondary white matter microstructure changes in chronic PI.

In addition, previous studies were mainly based on prior assumptions to explore particular white matter tracts (Zhang et al., 2015), and few studies have described the microstructural alterations at the whole-brain level. Tract-based spatial statistics (TBSS) is a relatively accurate and comprehensive method for evaluating changes in the whole-brain white matter microstructure (Smith et al., 2006). Compared with the traditional region of interest (ROI)-based analysis and voxel-based analysis (VBA) methods, it does not require the spatial smoothing of data before estimating the voxel-wise statistical analysis and does not establish any prior hypotheses (Luo et al., 2020; Zhang et al., 2021). This study aimed to investigate the correlations between DKI-derived diffusion and kurtosis parameters of abnormal white matter tracts and behavioral function in patients with chronic PI. Toward this goal, we constructed the white matter skeleton of the whole brain using TBSS based on DTI- and DKI-derived data. The white matter microstructure was first evaluated in patients with chronic PI by analyzing different diffusion and kurtosis parameters. Then, DTI-derived and DKI-derived parameters were combined to identify sensitive parameters for assessing white matter microstructure changes.

## Materials and Methods

### Subjects

**FIGURE 1:**
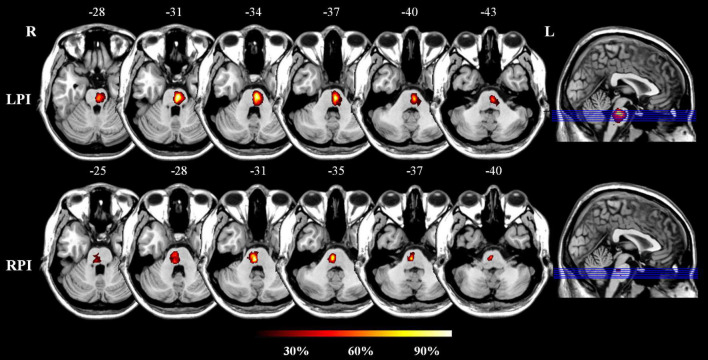
The lesion probability distribution maps in the LPI and RPI groups. The color bar represents the lesion probability. L and R, left and right hemisphere; LPI and RPI, left and right pontine infarction groups.

All subjects were recruited from the First Affiliated Hospital of Zhengzhou University and Tianjin Medical University General Hospital. This study was approved by the ethics committee, and informed consent was obtained from each subject. The inclusion criteria were as follows: (1) first-ever stroke; (2) age 40–80 years old; (3) a single lesion located in the unilateral pons; and (4) the examination time point was more than 6 months after infarction; and the exclusion criteria were as follows: (1) severe white matter hyperintensity with Fazekas scores > 1 and artery stenosis with a Ferguson score > 2; (2) a history of drug dependence or concomitant neuropsychiatric disorders; (3) severe systemic comorbidities, such as heart failure and cancer; and (4) contraindications for MRI. In total, 60 patients were included; of them, 33 and 27 patients had left PI (LPI) and right PI (RPI), respectively. In addition, 30 age-, sex-, and education-matched normal control (NC) subjects were recruited. The infarct lesions of LPI and RPI groups were manually delineated on the 3D high-resolution T1-weighted images using MRIcroN software.^1^ ROIs from all slices were concatenated to a volume of interest and then normalized in the Montreal Neurological Institute (MNI) space. The lesion masks of all patients were overlaid on the normalized template to create lesion distribution probability maps (Figure 1).

### MRI Data Acquisition

Magnetic resonance imaging was performed on the 3.0-Tesla MR scanner (Discovery MR 750, General Electric, Milwaukee, WI, United States). Tight but comfortable foam padding and earplugs were used to reduce head motion and scanner noise. The echo-planar imaging sequence was used to acquire diffusion images. DKI data were acquired with the following parameters: repetition time (TR) = 5,800 ms; echo time (TE) = 70.1 ms; fractional anisotropy (FA) = 90°; field of view (FOV) = 256 mm × 256 mm; matrix = 128 × 128; slice thickness = 3 mm; no gap; 48 axial slices; 25 encoding diffusion directions with two non-zero *b*-values (1,000 and 2,000 s/mm^2^) for each direction; 10 non-diffusion weighted images (*b* = 0 s/mm^2^).

### A Theoretical Model of Diffusion Tensor Imaging and Diffusion Kurtosis Imaging

The DTI model measured the motion of water molecules at the scale of micrometers, which is based on the assumption of Gaussian diffusion. The apparent diffusion coefficient (*D*_*app*_) of water molecules in each voxel can be calculated by linearly fitting a series of diffusion-weighted images to the following formula (1):

(1) $$\ln\frac{S\,\left(b\right)}{S\,\left(0\right)}=-b\,D_{a\,p\,p}$$

Where *S*(0) and *S*(*b*) represent the signal intensities at a *b*-value of 0 s/mm^2^ and *b*-values other than 0 s/mm^2^, respectively. Taking direction into consideration, the *D*_*app*_ can be extended to a second-order diffusion tensor. Then, the diffusion parameters, namely, the FA, mean diffusivity (MD), axial diffusivity (AD), and radial diffusivity (RD), were computed with the eigenvalue decomposition of the diffusion tensor.

Diffusion kurtosis imaging, as the extensive DTI model, takes into account the non-Gaussian properties of water diffusion and expresses the diffusion-weighted (DW) signals as the following formula (2):

(2) $$\ln\frac{S\,\left(b\right)}{S\,\left(0\right)}=-b\,D_{a\,p\,p}+\frac{1}{6}\,b^{2}\,D_{a\,p\,p}^{2}\,K_{a\,p\,p}$$

Where *K*_*app*_ is the apparent kurtosis. Similarly, the *K*_*app*_ can be extended to a fourth-order kurtosis tensor. In addition to the diffusion parameters, the DKI model provides complementary kurtosis parameters, such as the mean kurtosis (MK), axial kurtosis (AK), and radial kurtosis (RK), which can be calculated by using both diffusion and kurtosis tensors (Jensen and Helpern, 2010; Wu and Cheung, 2010).

### Imaging Processing

Diffusion kurtosis-weighted data were pre-processed and analyzed using the pipeline for analyzing brain diffusion images (PANDA) software (Cui et al., 2013). First, all diffusion images were converted to the Neuroimaging Informatics Technology Initiative (NIFTI) format. Second, the Brain Extraction Tool (BET) was applied to cutoff the non-brain tissue from the raw images and obtain the brain mask. Third, diffusion images were corrected for the eddy current distortions and head motion. Fourth, all DKI data (*b* = 0, 1,000 and 2,000 s/mm^2^) were used for DKI fitting and calculating diffusion parameters (FA_DKI_, MD_*DKI*,_ AD_DKI_, and RD_DKI_) and kurtosis parameters (MK_DKI_, AK_DKI_, and RK_DKI_) using Diffusional Kurtosis Estimator (DKE)^2^ with the constrained linear least-squares quadratic programming (CLLS-QP) algorithm (Tabesh et al., 2011). Fifth, only a part of the DKI data (*b* = 0 and 1,000 s/mm^2^) were employed for DTI fitting, and the DTI-derived parameters (FA_DTI_, MD_DTI_, AD_*DTI*,_ and RD_DTI_) were calculated by using a monoexponentially model. Sixth, FA maps (FA refers to FA_DTI_ when the DTI-derived parameters were processed, while it represents FA_DKI_ in the processing of DKI-derived parameters) of each subject were registered to the standard FMRIB58_FA template in the MNI space using the non-linear registration algorithm of the FNIRT tool. Seventh, the normalized individual FA maps were averaged to produce the group-level mean FA map, which was thinned to generate a mean FA skeleton under the threshold of 0.2 to exclude predominantly non-white matter voxels. Eighth, FA image of each subject was then projected onto the mean FA skeleton to obtain the synthetic FA skeleton images of all subjects. Ninth, other outcomes of diffusion and kurtosis parameters were projected onto the mean FA skeleton to obtain skeleton images of other parameters of all subjects. The anatomical locations of abnormal white matter tracts were denominated based on the JHU-ICBM-DTI-81 white matter atlas. The anatomical locations of abnormal white matter tracts were denominated based on the JHU-ICBM-DTI-81 white matter atlas.

For TBSS statistical analyses, a general linear model was established with age, gender, and years of education as covariates. Non-parametric permutation tests were performed with 5,000 permutations to explore intergroup differences in the diffusion and kurtosis parameters. The threshold-free cluster enhancement (TFCE) method was applied for multiple-comparison correction, and *P* < 0.01 was set as the significant threshold. In addition, to quantitatively compare the sensitivity between DTI and DKI techniques in detecting abnormal tissue microstructure, the percentage values of abnormal voxels identified by different parameters relative to the whole brain skeleton voxels were calculated. The whole analysis was accelerated and simplified through a cloud platform (Beijing Intelligent Brain Cloud, Inc.).^3^

### Behavioral Assessment

After MRI scans, each subject underwent neurobehavioral examinations, such as motor and cognitive functions. The Fugl-Meyer Assessment (FMA) was used to evaluate impaired motor function of all limbs in patients with PI. The test included 50 items measuring movements, reflexes, and coordination action about the shoulder, elbow, forearm, wrist, hand, hip, knee, and ankle. The total FMA score ranges from 0 (hemiplegia) to a maximum of 100 points (normal motor performance). Working memory, an important aspect of cognitive function, was assessed using a spatial 1-back test. The participates were instructed to respond as quickly and accurately as possible to determine any changes in the current position of the target white box in comparison with the previous one. The accuracy rate (ACC) and average reaction time (RT) for correct responses were recorded, with higher ACC scores and shorter RT defined to indicate better performance.

### Statistical Analysis

A two-sample *t*-test was used to compute the differences in age, years of education, and spatial 1-back scores between the NC and PI groups. Comparisons of sex were performed using the chi-square test. In addition, the DKI-derived diffusion and kurtosis parameters of abnormal white matter tracts showed intergroup differences after TBSS analyses were extracted. Spearman correlation analysis was used to assess the correlations between the mean parameter values and behavioral scores. All statistical analyses were performed using SPSS 21.0 software (version 21.0; SPSS, Inc., Chicago, IL, United States). These tests were two-tailed, and *P* < 0.05 was considered significant.

## Results

### Demographic and Behavioral Measures

There were no significant differences in demographic data, such as age, sex, and years of education, between the NC and PI groups. Lesion size and follow-up time also did not show statistical differences between LPI and RPI groups. Meanwhile, compared with the NC group, LPI and RPI groups showed behavioral dysfunctions with significantly lower FMT scores and more delayed RT in the spatial 1-back task (*P*_LPI_ = 0.02; *P*_RPI_ = 0.04). The characteristics of patients by the group are shown in Table 1.

**TABLE 1:** Demographic and clinical data of PI and NC groups.

Variable	PI (*n* = 60)		NC (*n* = 30)	*P*-values	
	LPI (*n* = 33)	RPI (*n* = 27)		LPI (*n* = 33)	RPI (*n* = 27)
Age, years	57.60 ± 5.68	57.03 ± 7.83	55.06 ± 6.48	*P*_LPI_ = 0.10	*P*_RPI_ = 0.30
Gender (male/female)	22/11	16/11	16/14	*P*_LPI_ = 0.31	*P*_RPI_ = 0.79
Years of education	10.63 ± 3.41	10.85 ± 3.42	10.33 ± 2.86	*P*_LPI_ = 0.71	*P*_RPI_ = 0.54
Lesion size (ml)	0.52 ± 0.51	0.34 ± 0.29	–	*P* = 0.09	
Timing of follow-up imaging (months)	9.66 ± 4.75	10.11 ± 5.47	–	*P* = 0.73	
Fugl-Meyer assessment	94.18 ± 14.34	95.35 ± 8.73	–	–	–
Spatial 1-back task					
*ACC* RT (ms)	0.90 ± 0.06 916.41 ± 225.51	0.91 ± 0.06 902.55 ± 250.66	0.92 ± 0.05 776.93 ± 189.82	*P*_LPI_ = 0.29 *P*_LPI_ = 0.01*	*P*_RPI_ = 0.68 *P*_RPI_ = 0.04*

*Continuous data were presented as the mean ± SD.*

LPI, left pontine infarction; RPI, right pontine infarction; NC, normal control; ACC, accuracy rate of the spatial 1-back task; RT, average reaction time for correct responses of the spatial 1-back task; P_LPI_, P-values of difference analyses between LPI and NC groups; P_RPI_, P-values of difference analyses between RPI and NC groups; P, P-values of the difference between LPI and RPI groups. *P < 0.05.

### Differences in Diffusion Tensor Imaging-Derived Diffusion Parameters

**FIGURE 2:**
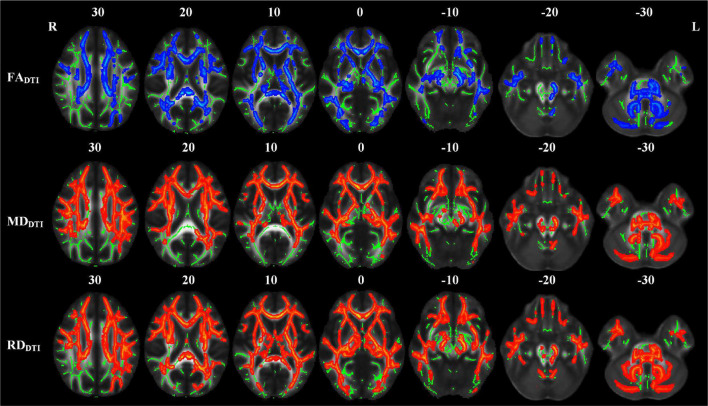
TBSS analyses showed abnormal white matter tracts in the LPI group with DTI-derived parameters. Red indicated white matter tracts with significantly increased parameter values and blue indicated white matter tracts with significantly decreased parameter values. Green represented the mean FA skeleton of all subjects. L and R, left and right; FA, fractional anisotropy; MD, mean diffusivity; RD, radial diffusivity; TBSS, tract-based spatial statistics; DTI, diffusion tensor imaging.

Compared with the NC group, the LPI group showed significant changes in white matter microstructure in extensive bilateral supra- and infratentorial regions (Figure 2 and Table 2). In the LPI group, DTI_TBSS analysis showed that FA_DTI_ was significantly decreased while MD_DTI_ and RD_DTI_ were increased in some white matter tracts, namely, the middle cerebellar peduncle (MCP), pontine crossing tract (PCT), genu of corpus callosum (Genu of CC), body of corpus callosum (Body of CC), splenium of corpus callosum (Splenium of CC), fornix (FX), bilateral medial lemniscus (ML_R, ML_L), inferior cerebellar peduncle (ICP_R, ICP_L), superior cerebellar peduncle (SCP_R, SCP_L), cerebral peduncle (CP_R, CP_L), anterior limb of internal capsule (ALIC_R, ALIC_L), posterior limb of internal capsule (PLIC_R, PLIC_L), retrolenticular part of internal capsule (RLIC_R, RLIC_L), anterior corona radiata (ACR_R, ACR_L), superior corona radiata (SCR_R, SCR_L), posterior corona radiata (PCR_R, PCR_L), posterior thalamic radiation (PTR_R, PTR_L), sagittal stratum (SS_R, SS_L), external capsule (EC_R, EC_L), stria terminalis (ST_R, ST_L), superior longitudinal fasciculus (SLF_R, SLF_L), uncinate fasciculus (UNC_R, UNC_L), left corticospinal tract (CST_L), right superior fronto-occipital fasciculus (SFO_R), and right tapetum (TAP_R). Some white matter tracts only showed significantly increased MD_DTI_ and RD_DTI_ in the right corticospinal tract (CST_R) and bilateral cingulate gyrus (CG_R, CG_L) only with increased RD_DTI_. The AD_DTI_ results were not a significant difference between the NC and LPI groups. With respect to sensitivity, FA_DTI_, MD_DTI_, and RD_DTI_ could identify 27.3, 40.0, and 46.0% of abnormal white matter voxels in the whole brain white matter skeleton, respectively.

**TABLE 2:** Abnormal white matter fibers derived DTI different parameters in LPI group compared with NC group.

Group	Parameters	Cluster index	Cluster size (voxels)	MNI coordinates			Minimally *P*-values	White matter tracts
LPI	FA_DTI_	1	116	50	96	54	0.01	SS_R
		2	498	130	126	94	0.008	SLF_L
		3	1,904	50	128	40	0.009	CP_R; PLIC_R; RLIC_R; SS_R; ST_R; UNC_R
		4	35,587	91	88	21	0.001	MCP, PCT, Genu of CC; Body of CC; Splenium of CC; FX; CST_L; ML_R; ML_L; ICP_R; ICP_L; SCP_R; SCP_L; CP_L; ALIC_R; ALIC_L; PLIC_R; PLIC_L; RLIC_L; ACR_R; ACR_L; SCR_R; SCR_L; PCR_R; PCR_L; PTR_R; PTR_L; SS_R; SS_L; EC_R; EC_L; ST_L; SLF_R; SLF_L; SFO_R; UNC_L; TAP_R.
	MD_DTI_	1	55,860	112	164	69	0.001	MCP, PCT, Genu of CC; Body of CC; Splenium of CC; FX; CST_R; CST_L; ML_R; ML_L; ICP_R; ICP_L; SCP_R; SCP_L; CP_R; CP_L; ALIC_R; ALIC_L; PLIC_R; PLIC_L; RLIC_R; RLIC_L; ACR_R; ACR_L; SCR_R; SCR_L; PCR_R; PCR_L; PTR_R; PTR_L; SS_R; SS_L; EC_R; EC_L; ST_R; ST_L; SLF_R; SLF_L; SFO_R; UNC_R; UNC_L; TAP_R.
	RD_DTI_	1	64,218	91	88	21	0.001	MCP, PCT, Genu of CC; Body of CC; Splenium of CC; FX; CST_R; CST_L; ML_R; ML_L; ICP_R; ICP_L; SCP_R; SCP_L; CP_R; CP_L; ALIC_R; ALIC_L; PLIC_R; PLIC_L; RLIC_R; RLIC_L; ACR_R; ACR_L; SCR_R; SCR_L; PCR_R; PCR_L; PTR_R; PTR_L; SS_R; SS_L; EC_R; EC_L;CG_R; CG_L; ST_R; ST_L; SLF_R; SLF_L; SFO_R; UNC_R; UNC_L; TAP_R.

*LPI, left pontine infarction; FA, fractional anisotropy; MD, mean diffusivity; RD, radial diffusivity; MCP, Middle cerebellar peduncle; PCT, Pontine crossing tract (a part of MCP); Genu of CC, Genu of corpus callosum; Body of CC, Body of corpus callosum; Splenium of CC, Splenium of corpus callosum; FX, Fornix (column and body of fornix); CST_R, Corticospinal tract R; CST_L, Corticospinal tract L; ML_R, Medial lemniscus R; ML_L, Medial lemniscus L; ICP_R, Inferior cerebellar peduncle R; ICP_L, Inferior cerebellar peduncle L; SCP_R, Superior cerebellar peduncle R; SCP_L, Superior cerebellar peduncle L; CP_R, Cerebral peduncle R; CP_L, Cerebral peduncle L; ALIC_R, Anterior limb of internal capsule R; ALIC_L, Anterior limb of internal capsule L; PLIC_R, Posterior limb of internal capsule R; PLIC_L, Posterior limb of internal capsule L; RLIC_R, Retrolenticular part of internal capsule R; RLIC_L, Retrolenticular part of internal capsule L; ACR_R, Anterior corona radiata R; ACR_L, Anterior corona radiata L; SCR_R, Superior corona radiata R; SCR_L, Superior corona radiata L; PCR_R, Posterior corona radiata R; PCR_L, Posterior corona radiata L; PTR_R, Posterior thalamic radiation (include optic radiation) R; PTR_L, Posterior thalamic radiation (include optic radiation) L; SS_R, Sagittal stratum (include inferior longitudinal fasciculus and inferior fronto_occipital fasciculus) R; SS_L, Sagittal stratum (include inferior longitudinal fasciculus and inferior fronto_occipital fasciculus) L; EC_R, External capsule R; EC_L, External capsule L; CG_R, Cingulum (cingulate gyrus) R; CG_L, Cingulum (cingulate gyrus) L; CH_R, Cingulum (hippocampus) R; CH_L, Cingulum (hippocampus) L; ST_R, Fornix (cres) / Stria terminalis R; ST_L, Fornix (cres) / Stria terminalis L; SLF_R, Superior longitudinal fasciculus R; SLF_L, Superior longitudinal fasciculus L; SFO_R, Superior fronto_occipital fasciculus (could be a part of anterior internal capsule) R; SFO_L, Superior fronto_occipital fasciculus (could be a part of anterior internal capsule) L; UNC_R, Uncinate fasciculus R; UNC_L, Uncinate fasciculus L; TAP_R, Tapetum R; TAP_L, Tapetum L.*

**FIGURE 3:**
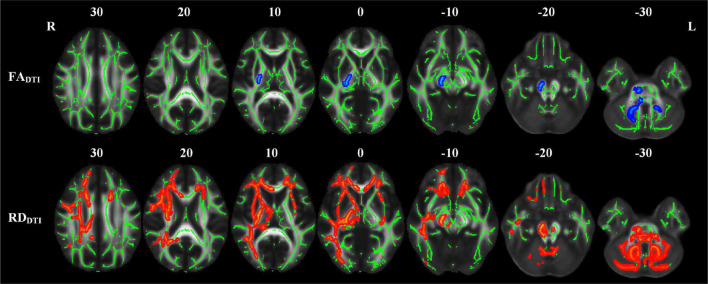
TBSS analyses showed abnormal white matter tracts in the RPI group with DTI-derived parameters. Red indicated white matter tracts with significantly increased parameter values and blue indicated white matter tracts with significantly decreased parameter values. Green represented the mean FA skeleton of all subjects. L and R, left and right; FA, fractional anisotropy; MD, mean diffusivity; RD, radial diffusivity; TBSS, tract-based spatial statistics; DTI, diffusion tensor imaging.

The white matter tracts with significant intergroup differences in the RPI group are shown in Figure 3 and Table 3. These abnormal white matter tracts with decreased FA_DTI_ and increased RD_DTI_ were mainly in the MCP, PCT, CST_R, CST_L, ML_R, ICP_R, ICP_L, SCP_R, CP_R, PLIC_R, and RLIC_R. In addition, tracts with only significantly increased RD_DTI_ were found in Genu of CC, Body of CC, Splenium of CC, FX, ML_L, SCP_L, ALIC_R, ACR_R, ACR_L, SCR_R, SCR_L, PCR_R, PTR_R, SS_R, EC_R, EC_L, CG_R, ST_R, SLF_R, and SFO_R. There were no significant differences in MD_DTI_ and AD_DTI_ between the NC and RPI groups. The sensitivity of FA_*DTI*,_ RD_DTI_ showed that they could identify 1.7 and 19.7% of abnormal white matter voxels in the whole brain white matter skeleton, respectively.

**TABLE 3:** Abnormal white matter fibers derived DTI different parameters in RPI group compared with NC group.

Group	Parameters	Cluster index	Cluster size (voxels)	MNI coordinates			Minimally *P*-values	White matter tracts
RPI	FA_DTI_	1	176	103	83	37	0.0008	MCP, ICP_L
		2	2,226	86	91	25	0.001	MCP; PCT; CST_R; CST_L; ML_R; ICP_R; SCP_R; CP_R; PLIC_R; RLIC_R
	RD_DTI_	1	25,053	89	87	18	0.001	MCP, PCT, Genu of CC; Body of CC; Splenium of CC; FX; CST_R; CST_L; ML_R; ML_L; ICP_R; ICP_L; SCP_R; SCP_L; CP_R; ALIC_R; PLIC_R; RLIC_R; ACR_R; SCR_R; PCR_R; PTR_R; SS_R; EC_R; CG_R; ST_R; SLF_R; SFO_R;
		2	2,371	116	157	80	0.008	Genu of CC; Body of CC; ACR_L; SCR_L; EC_L

*RPI, right pontine infarction; FA, fractional anisotropy; RD, radial diffusivity; MCP, Middle cerebellar peduncle; PCT, Pontine crossing tract (a part of MCP); Genu of CC, Genu of corpus callosum; Body of CC, Body of corpus callosum; Splenium of CC, Splenium of corpus callosum; FX, Fornix (column and body of fornix); CST_R, Corticospinal tract R; CST_L, Corticospinal tract L; ML_R, Medial lemniscus R; ML_L, Medial lemniscus L; ICP_R, Inferior cerebellar peduncle R; ICP_L, Inferior cerebellar peduncle L; SCP_R, Superior cerebellar peduncle R; SCP_L, Superior cerebellar peduncle L; CP_R, Cerebral peduncle R; ALIC_R, Anterior limb of internal capsule R; PLIC_R, Posterior limb of internal capsule R; RLIC_R, Retrolenticular part of internal capsule R; ACR_R, Anterior corona radiata R; ACR_L, Anterior corona radiata L; SCR_R, Superior corona radiata R; SCR_L, Superior corona radiata L; PCR_R, Posterior corona radiata R; PTR_R, Posterior thalamic radiation (include optic radiation) R; SS_R, Sagittal stratum (include inferior longitudinal fasciculus and inferior fronto_occipital fasciculus) R; EC_R, External capsule R; EC_L, External capsule L;CG_R, Cingulum (cingulate gyrus) R; ST_R, Fornix (cres) / Stria terminalis R; SLF_R, Superior longitudinal fasciculus R; SFO_R, Superior fronto_occipital fasciculus (could be a part of anterior internal capsule) R.*

### Differences in Diffusion Kurtosis Imaging-Derived Diffusion and Kurtosis Parameters

**FIGURE 4:**
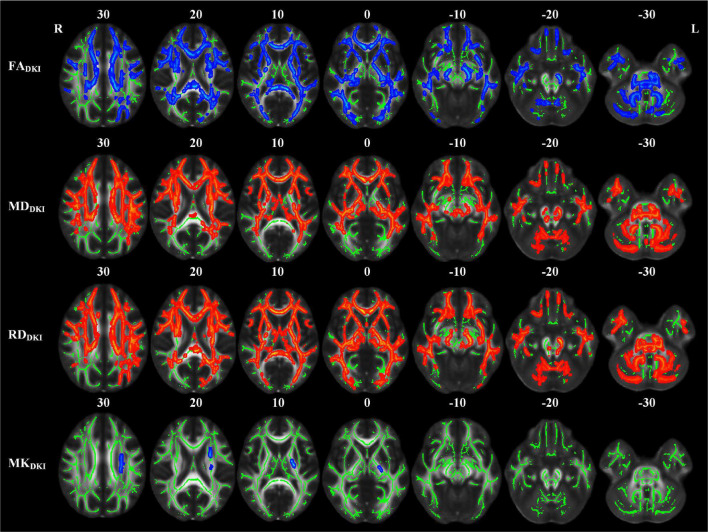
TBSS analyses showed abnormal white matter tracts in the LPI group with DKI-derived parameters. Red indicated white matter tracts with significantly increased parameter values and blue indicated white matter tracts with significantly decreased parameter values. Green represented the mean FA skeleton of all subjects. L and R, left and right; FA, fractional anisotropy; MD, mean diffusivity; RD, radial diffusivity; TBSS, tract-based spatial statistics; DKI, diffusion kurtosis imaging.

The abnormal white matter tracts with significant intergroup differences in DKI-derived diffusion and kurtosis parameters in the LPI group are shown in Figure 4 and Table 4. Compared with the NC group, the LPI group showed significantly impaired white matter microstructures in extensive bilateral supra- and infratentorial regions. These impairments were characterized by decreased FA_DKI_ and increased MD_DKI_ and RD_DKI_ in MCP, PCT, Genu of CC, Body of CC, Splenium of CC, FX, CST_R, CST_L, ML_R, ML_L, ICP_R, ICP_L, SCP_R, SCP_L, CP_R, CP_L, ALIC_R, ALIC_L, PLIC_R, PLIC_L, RLIC_R, RLIC_L, ACR_R, ACR_L, SCR_R, SCR_L, PCR_R, PCR_L, PTR_R, PTR_L, SS_R, SS_L, EC_R, EC_L, ST_R, ST_L, SLF_R, SLF_L, SFO_R, and UNC_L. Abnormal white matter tracts with only significantly decreased FA_DKI_ and increased RD_DKI_ were found in CG_R, CG_L, UNC_R, and TAP_R. For kurtosis parameters, MK_DKI_ was significantly decreased in the PLIC_L, ACR_L, SCR_L, PCR_L, and SLF_L. However, there were no significant differences in other diffusion and kurtosis parameters, such as AD_DKI_, AK_DKI_, and RK_DKI_. With respect to sensitivity, FA_DKI_, MD_DKI_, RD_DKI_, and MK_DKI_ could identify 34.6, 40.2, 47.9, and 0.58% of abnormal white matter voxels in the whole brain white matter skeleton, respectively.

**TABLE 4:** Abnormal white matter tracts derived from DKI different parameters in the LPI group.

Group	Parameters	Cluster index	Cluster size (voxels)	MNI coordinates			Minimally *P*-values	White matter tracts
LPI	FA_DKI_	1	47,944	91	0.001	23	0.001	MCP, PCT, Genu of CC, Body of CC, Splenium of CC, FX, CST_R, CST_L, ML_R, ML_L, ICP_R, ICP_L, SCP_R, SCP_L, CP_R, CP_L, ALIC_R, ALIC_L, PLIC_R, PLIC_L, RLIC_R, RLIC_L, ACR_R, ACR_L, SCR_R, SCR_L, PCR_R, PCR_L, PTR_R, PTR_L, SS_R, SS_L, EC_R, EC_L, CG_R, CG_L, ST_R, ST_L, SLF_R, SLF_L, SFO_R, UNC_R, UNC_L, TAP_R
	MD_DKI_	1	55,715	99	156	156	0.001	MCP, PCT, Genu of CC, Body of CC, Splenium of CC, FX, CST_R, CST_L, ML_R, ML_L, ICP_R, ICP_L, SCP_R, SCP_L, CP_R, CP_L, ALIC_R, ALIC_L, PLIC_R, PLIC_L, RLIC_R, RLIC_L, ACR_R, ACR_L, SCR_R, SCR_L, PCR_R, PCR_L, PTR_R, PTR_L, SS_R, SS_L, EC_R, EC_L, ST_R, ST_L, SLF_R, SLF_L, SFO_R, UNC_L
	RD_DKI_	1	66462	96	76	23	0.001	MCP, PCT, Genu of CC, Body of CC, Splenium of CC, FX, CST_R, CST_L, ML_R, ML_L, ICP_R, ICP_L, SCP_R, SCP_L, CP_R, CP_L, ALIC_R, ALIC_L, PLIC_R, PLIC_L, RLIC_R, RLIC_L, ACR_R, ACR_L, SCR_R, SCR_L, PCR_R, PCR_L, PTR_R, PTR_L, SS_R, SS_L, EC_R, EC_L, CG_R, CG_L, ST_R, ST_L, SLF_R, SLF_L, SFO_R, UNC_R, UNC_L, TAP_R
	MK _DKI_	1	191	112	109	72	0.009	PLIC_L
		2	617	111	98	115	0.005	ACR_L, SCR_L, PCR_L, SLF_L

*LPI, left pontine infarction; FA, fractional anisotropy; MD, mean diffusivity; RD, radial diffusivity; MK, mean kurtosis; DKI, Diffusion Kurtosis Imaging; MCP, Middle cerebellar peduncle; PCT, Pontine crossing tract (a part of MCP); Genu of CC, Genu of corpus callosum; Body of CC, Body of corpus callosum; Splenium of CC, Splenium of corpus callosum; FX, Fornix (column and body of fornix); CST_R, Corticospinal tract R; CST_L, Corticospinal tract L; ML_R, Medial lemniscus R; ML_L, Medial lemniscus L; ICP_R, Inferior cerebellar peduncle R; ICP_L, Inferior cerebellar peduncle L; SCP_R, Superior cerebellar peduncle R; SCP_L, Superior cerebellar peduncle L; CP_R, Cerebral peduncle R; CP_L, Cerebral peduncle L; ALIC_R, Anterior limb of internal capsule R; ALIC_L, Anterior limb of internal capsule L; PLIC_R, Posterior limb of internal capsule R; PLIC_L, Posterior limb of internal capsule L; RLIC_R, Retrolenticular part of internal capsule R; RLIC_L, Retrolenticular part of internal capsule L; ACR_R, Anterior corona radiata R; ACR_L, Anterior corona radiata L; SCR_R, Superior corona radiata R; SCR_L, Superior corona radiata L; PCR_R, Posterior corona radiata R; PCR_L, Posterior corona radiata L; PTR_R, Posterior thalamic radiation (include optic radiation) R; PTR_L, Posterior thalamic radiation (include optic radiation) L; SS_R, Sagittal stratum (include inferior longitudinal fasciculus and inferior fronto_occipital fasciculus) R; SS_L, Sagittal stratum (include inferior longitudinal fasciculus and inferior fronto_occipital fasciculus) L; EC_R, External capsule R; EC_L, External capsule L; CG_R, Cingulum (cingulate gyrus) R; CG_L, Cingulum (cingulate gyrus) L; ST_R, Fornix (cres) / Stria terminalis R; ST_L, Fornix (cres) / Stria terminalis L; SLF_R, Superior longitudinal fasciculus R; SLF_L, Superior longitudinal fasciculus L; SFO_R, Superior fronto_occipital fasciculus (could be a part of anterior internal capsule) R; UNC_R, Uncinate fasciculus R; UNC_L, Uncinate fasciculus L; TAP_R, Tapetum R.*

**FIGURE 5:**
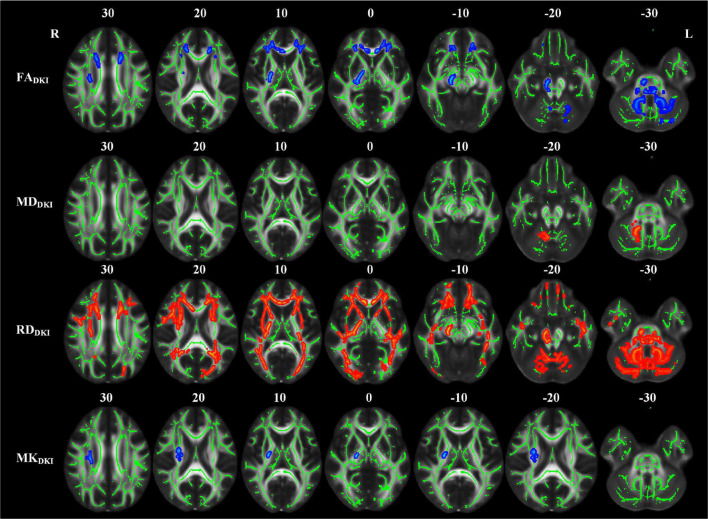
TBSS analyses showed abnormal white matter tracts in the RPI group with DKI-derived parameters. Red indicated white matter tracts with significantly increased parameter values and blue indicated white matter tracts with significantly decreased parameter values. Green represented the mean FA skeleton of all subjects. L and R, left and right; FA, fractional anisotropy; MD, mean diffusivity; RD, radial diffusivity, TBSS, tract-based spatial statistics; DKI, diffusion kurtosis imaging.

Compared with the NC group, the RPI group also showed extensive white matter microstructure changes (Figure 5 and Table 5). In these abnormal white matter tracts, MCP and ICP_R showed significantly decreased FA_DKI_ and increased MD_DKI_ and RD_DKI_. Some abnormal white matter tracts only presented with decreased FA_DKI_ and increased RD_DKI_ in PCT, Genu of CC, Body of CC, ML_R, ML_L, ICP_R, ICP_L, SCP_R, SCP_L, ACR_R, ACR_L, SCR_R, SCR_L, CST_R, CP_R, ALIC_R, PLIC_R, RLIC_R, EC_R, and SLF_R. Moreover, abnormal white matter tracts only with significantly increased RD_DKI_ were found in the splenium of CC, PCR_R, PCR_L, PTR_R, PTR_L, SS_R, SS_L, ST_R, ST_L, TAP_R, TAP_L, CST_L, ALIC_L, PLIC_L, RLIC_L, EC_L, SLF_L, SFO_R, and UNC_L. For kurtosis parameters, MK_DKI_ was significantly decreased in PLIC_R, SCR_R, PCR_R, EC_R, and SLF_R. Other parameters, including AD_DKI_, AK_DKI_, and RK_DKI_, did not show significant differences. With respect to sensitivity, FA_DKI_, MD_DKI_, RD_DKI_, and MK_DKI_ could identify 6.9, 0.6, 24.1, and 0.56% of abnormal white matter voxels in the whole brain white matter skeleton, respectively.

**TABLE 5:** Abnormal white matter fibers derived DKI different parameters in the RPI group.

Group	Parameters	Cluster index	Cluster size (voxels)	MNI coordinates			Minimally *P*-values	White matter tracts
RPI	FA_DKI_	1	1,475	111	156	65	0.007	Genu of CC, Body of CC, ACR_L, SCR_L
		2	1,593	63	160	74	0.007	Genu of CC, Body of CC, ACR_R, SCR_R
		3	1,775	70	102	119	0.006	SCR_R, SLF_R
		4	4,829	83	95	30	0.001	MCP, PCT, CST_R, ML_R, ML_L, ICP_R, ICP_L, SCP_R, SCP_L, CP_R, ALIC_R, PLIC_R, RLIC_R, EC_R
	MD _DKI_	1	895	75	85	35	0.007	MCP, ICP_R
	RD_DKI_	1	33,618	74	82	38	0.001	MCP, PCT, Genu of CC, Body of CC, Splenium of CC, CST_R, CST_L, ML_R, ML_L, ICP_R, ICP_L, SCP_R, SCP_L, CP_R, ALIC_R, ALIC_L, PLIC_R, PLIC_L, RLIC_R, RLIC_L, ACR_R, ACR_L, SCR_R, SCR_L, PCR_R, PCR_L, PTR_R, PTR_L, SS_R, SS_L, EC_R, EC_L, ST_R, ST_L, SLF_R, SLF_L, SFO_R, UNC_L, TAP_R, TAP_L
	MK _DKI_	1	781	63	110	99	0.997	PLIC_R, SCR_R, PCR_R, EC_R, SLF_R

*RPI, right pontine infarction; FA, fractional anisotropy; MD, mean diffusivity; RD, radial diffusivity; MK, mean kurtosis; DKI, Diffusion Kurtosis Imaging; MCP, Middle cerebellar peduncle; PCT, Pontine crossing tract (a part of MCP); Genu of CC, Genu of corpus callosum; Body of CC, Body of corpus callosum; Splenium of CC, Splenium of corpus callosum; CST_R, Corticospinal tract R; CST_L, Corticospinal tract L; ML_R, Medial lemniscus R; ML_L, Medial lemniscus L; ICP_R, Inferior cerebellar peduncle R; ICP_L, Inferior cerebellar peduncle L; SCP_R, Superior cerebellar peduncle R; SCP_L, Superior cerebellar peduncle L; CP_R, Cerebral peduncle R; ALIC_R, Anterior limb of internal capsule R; ALIC_L, Anterior limb of internal capsule L; PLIC_R, Posterior limb of internal capsule R; PLIC_L, Posterior limb of internal capsule L; RLIC_R, Retrolenticular part of internal capsule R; RLIC_L, Retrolenticular part of internal capsule L; ACR_R, Anterior corona radiata R; ACR_L, Anterior corona radiata L; SCR_R, Superior corona radiata R; SCR_L, Superior corona radiata L; PCR_R, Posterior corona radiata R; PCR_L, Posterior corona radiata L; PTR_R, Posterior thalamic radiation (include optic radiation) R; PTR_L, Posterior thalamic radiation (include optic radiation) L; SS_R, Sagittal stratum (include inferior longitudinal fasciculus and inferior fronto_occipital fasciculus) R; SS_L, Sagittal stratum (include inferior longitudinal fasciculus and inferior fronto_occipital fasciculus) L; EC_R, External capsule R; EC_L, External capsule L; ST_R, Fornix (cres) / Stria terminalis R; ST_L, Fornix (cres) / Stria terminalis L; SLF_R, Superior longitudinal fasciculus R; SLF_L, Superior longitudinal fasciculus L; SFO_R, Superior fronto_occipital fasciculus (could be a part of anterior internal capsule) R; UNC_L, Uncinate fasciculus L; TAP_R, Tapetum R; TAP_L, Tapetum.*

### Correlations Between Diffusion Kurtosis Imaging-Derived Parameters and Behavioral Scores

The correlations between diffusion and kurtosis values of abnormal white matter tracts and behavioral functions in patients with PI are shown in Table 6. Decreased FA_DKI_ and MK_DKI_ values and increased MD_DKI_ and RD_DKI_ values were correlated with motor and cognition function. In the LPI group, decreased FA_DKI_ values in MCP, PCT, CST_L, ICP_R, and CP_L were positively correlated with FMT scores. Meanwhile, decreased FA_DKI_ values in Genu of CC, PCR_L, and SLF_R were positively correlated with the accuracy rate of the spatial-1back (SPA_ACC) scores. Decreased FA_DKI_ values in CST_L were negatively correlated with SPA_ACC scores. Whereas decreased FA_DKI_ in PCT, CST_L, and CG_R were negatively correlated with the average reaction time for correct responses of spatial-1 back (SPA_RT) scores. Meanwhile, increased MD_DKI_ values in PCT and CST_L were negatively correlated with FMT scores and positively correlated with SPA_RT values. Increased MD_DKI_ values in Genu of CC, ACR_R, and ACR_L were negatively correlated with SPA_ACC scores. On the other hand, decreased MK_DKI_ values in SCR_L and SLF_L were positively correlated with SPA_ACC scores. Increased RD_DKI_ in MCP, PCT, CST_L, and CP_L were negatively correlated with FMT scores and increased RD_DKI_ in Genu of CC, ACR_R, and ACR_L were negatively correlated with SPA_ACC scores. Increased RD_DKI_ in PCT and CST_L were positively correlated with SPA_RT values.

**TABLE 6:** Correlations between DKI parameters and behavioral scores in PI patients.

Group	Behavioral test	Correlated white matter fibers and parameters	*R*-values	*P-*values
LPI	FMT	FA_DKI_ values of MCP	0.396	0.022
		FA_DKI_ values of PCT	0.352	0.045
		FA_DKI_ values of CST_L	0.561	0.001
		FA_DKI_ values of ICP_R	0.366	0.036
		FA_DKI_ values of CP_L	0.458	0.007
		MD_DKI_ values of PCT	–0.415	0.016
		MD_DKI_ values of CST_L	–0.481	0.005
		RD_DKI_ values of MCP	–0.387	0.026
		RD_DKI_ values of PCT	–0.462	0.007
		RD_DKI_ values of CST_L	–0.473	0.005
		RD_DKI_ values of CP_L	–0.366	0.036
	SPA-ACC	FA_DKI_ values of Genu of CC	0.386	0.027
		FA_DKI_ values of CST_L	–0.390	0.025
		FA_DKI_ values of PCR_L	0.355	0.043
		FA_DKI_ values of SLF_R	0.532	0.001
		MD_DKI_ values of Genu of CC	–0.362	0.038
		MD_DKI_ values of ACR_R	–0.404	0.020
		MD_DKI_ values of ACR_L	–0.391	0.025
		MK_DKI_ values of SCR_L	0.415	0.016
		MK_DKI_ values of SLF_L	0.493	0.004
		RD_DKI_ values of Genu of CC	–0.433	0.012
		RD_DKI_ values of ACR_R	–0.386	0.037
		RD_DKI_ values of ACR_L	–0.430	0.013
	SPA-RT	FA_DKI_ values of PCT	–0.412	0.017
		FA_DKI_ values of CST_L	–0.438	0.011
		FA_DKI_ values of CG_R	–0.368	0.034
		MD_DKI_ values of PCT	0.354	0.043
		MD_DKI_ values of CST_L	0.404	0.020
		RD_DKI_ values of PCT	0.381	0.029
		RD_DKI_ values of CST_L	0.415	0.016
RPI	FMT	FA_DKI_ values of ACR_R	0.394	0.047
		FA_DKI_ values of ACR_L	0.499	0.009
		RD_DKI_ values of PCT	0.455	0.019
	SPA-ACC	MK_DKI_ values of EC_R	0.425	0.034
		RD_DKI_ values of SLF_L	–0.499	0.011

LPI and RPI, left and right pontine infarction; FA, fractional anisotropy; MD, mean diffusivity; RD, radial diffusivity; MK, mean kurtosis; MCP, Middle cerebellar peduncle; PCT, Pontine crossing tract (a part of MCP); CST_L, Corticospinal tract L; ICP_R, Inferior cerebellar peduncle R; CP_L, Cerebral peduncle L; SLF_R, Superior longitudinal fasciculus R; Genu of CC, Genu of corpus callosum; ACR_R, Anterior corona radiata R; ACR_L, Anterior corona radiata L; SCR_L, Superior corona radiata L; CG_R, Cingulum (cingulate gyrus) R; SPA-ACC, The accuracy rate of the spatial-1 back; SPA-RT, The average reaction time for correct responses of spatial-1 back (SPA_RT).

In the RPI group, decreased FA_DKI_ values in ACR_R and ACR_L were positively correlated with FMT scores. Increased RD_DKI_ in PCT was positively correlated with the FMT scores. Decreased MK_DKI_ in EC_R was positively correlated with SPA_ACC scores, and increased RD_DKI_ in SLF_L was negatively correlated with SPA_ACC scores.

## Discussion

Data on secondary white matter microstructure changes in the whole-brain level to chronic PI are currently rare. In this study, we used both DTI- and DKI-TBSS analyses to explore whole-brain white matter microstructural changes in patients with chronic PI. The results found extensive perilesional and remote white matter microstructures changes in patients with chronic PI and decreased FA was primarily due to increased RD. In addition, compared with DTI-TBSS findings, DKI-derived parameters could provide more comprehensive information for understanding the neuropathology of the chronic PI and are more sensitive in detecting impaired white matter microstructure. Moreover, DKI-derived diffusion and kurtosis parameters of abnormal white matter tracts were significantly correlated with behavioral scores, such as motor and cognition function, in the PI groups. These findings indicate that extensive impairments in white matter microstructures maybe a potential mechanism underlying behavioral dysfunction in chronic PI.

The current study documented extensive white matter microstructures impairment in chronic PI with significantly decreased FA, increased MD and RD, which not only included perilesional white matter tracts but also in remote ipsilateral, contralateral, and commissural white matter tracts. The human brain is a complex network that coordinates intra- and inter-subnetwork connectivity to modulate various physiological functions (Wang et al., 2014). Global or local tissue destruction with cortical atrophy and abnormal functional connectivity has been demonstrated in multiple subnetworks after stroke (Wang et al., 2020; Wei et al., 2020a). Therefore, the extensive white matter degeneration maybe the underlying neural basis and may support the concept of stroke as a disorder of global network dysconnectivity. Previous studies have mainly focused on specific white matter fibers, such as the CST after PI (Zhang et al., 2015), but this local microscopic measurement maybe incomprehensive. In this study, we demonstrated extensive white matter tracts disruption, suggested secondary macroscopic and microscopic structural changes in the tissue after PI. Therefore, white matter damage in global brain tissue needs to be considered when evaluating pathophysiological change and the therapeutic efficacy. Moreover, different diffusion and kurtosis parameters represent various histological features. FA measures the mean anisotropy of water molecules and reflects the distribution of AD and RD changes (Kamiya et al., 2016). MD indicates water content or extracellular fluid (Phillipou et al., 2018). AD and RD indirectly represent axon and myelin sheath integrity, respectively (Luo et al., 2020). Impaired white matter fibers showed significant changes in FA and RD but not in AD, which suggested decreased FA values are mainly caused by the increased RD and that the white matter microstructure disruption in chronic PI is mainly caused by impaired myelin sheath integrity. This finding is consistent with those of a previous histological report of widespread myelin disruption in the chronic stage after stroke (Borich et al., 2013).

Kurtosis parameters are sensitive indicators for characterizing tissue complexity and reflecting the heterogeneity of the tissue microenvironment, which could be influenced by the myelin, axonal, neural density, and glial proliferation (Jensen and Helpern, 2010; Zhang et al., 2017). MK reflects the mean diffuse kurtosis of water molecules in all directions. AK and RK refer to the kurtosis information that diffuses parallel and perpendicular to axon direction, respectively (Zhuo et al., 2012). In contrast to the widespread white matter tracts abnormalities in diffusion parameters results, kurtosis parameters only showed significantly decreased MK mainly in the ipsilesional white matter fibers with complex fiber arrangement (e.g., the juxtacortical white matter and corona radiata). This was consistent with previous observations that diffusion parameters were sensitive to detecting abnormal white matter regions with coherent fiber arrangement, and kurtosis parameters were sensitive to exploring abnormalities in white matter regions with complex fiber arrangement (Li et al., 2018). Experimental stroke studies have indicated that kurtosis parameters exhibited spatiotemporal dynamics that showed significantly abnormal changes in hyperacute and acute ischemia stage and started to pseudo-normalize at chronic stage (Hui et al., 2012). Moreover, although extensively abnormal diffusion parameters indicate disrupted myelin and increased extracellular water content (Phillipou et al., 2018), the global distribution of activated microglia in chronic stroke may maintain tissue complexity (Shi et al., 2019). These may account for less dissimilarity in kurtosis results. A similar phenomenon has been observed in other neurological diseases, such as Moyamoya and Alzheimer’s disease (Kazumata et al., 2016; Raj et al., 2021). Therefore, diffusion and kurtosis parameters could be complementary, and their combination could provide a more detailed description of neuropathological alterations in chronic PI. Our findings provide a theoretical basis for selecting more sensitive diffusion and kurtosis parameters to assess post-PI tissue microstructure of different stages in specific white matter regions.

We also compared the consistencies and differences in DTI- and DKI-derived parameters in TBSS analyses. Compared with DTI-derived diffusion parameters, DKI could identify more and larger regions of white matter microstructure changes. Particularly, DTI-derived MD in the RPI group did not show any significantly abnormal tracts, whereas significantly increased MD was found in MCP and ICP_R from DKI results. In addition, sensitivity analysis showed that DKI identified a higher percentage of abnormal white matter voxels than did DTI, consistent with previous findings on neuropsychiatric diseases (Zhu et al., 2015; Yang et al., 2021). The DTI-derived diffusion parameters are estimated to character monoexponential signal decay, which is not adequate due to the restricted diffusion environment. However, the diffusivity derived from DKI is calculated by using a second-order polynomial modal to character non-monoexponential signal decay (Cheung et al., 2009). In addition, the diffusivity of conventional DTI modal indirectly reflects a combined effect of the diffusivity and kurtosis derived from DKI (Cheung et al., 2009), and the different effects on DKI-derived diffusion and kurtosis parameters from chronic stroke could lead to less reduction in DTI-derived diffusivity. Therefore, DKI-derived parameters could provide more abundant and more precise information for investigating the neuronal mechanisms underlying behavioral dysfunctions in patients with PI.

Correlation analyses between DKI-derived parameters and behavioral scores in the current study suggested that abnormal parameters in the CST, CR, CP, PCT, and MCP were correlated with impaired motor function. Meanwhile, abnormal parameters in the Genu of CC, PCT, CST, CR, and SLF were correlated with working memory scores. CST is considered to be restricted to the motor and sensory domains, but some studies have indicated that disrupted CST could also result in lower action speed during cognitive tasks and, in turn, affect the cognitive domain (Puy et al., 2018). Moreover, CR, CP, PCT, and MCP are all important components of the cortico-ponto-cerebellar (CPCT) pathway. The CPCT pathway acts as a vital afferent fiber from the cerebral cortex to the contralateral MCP and is involved in the coordination of movement and higher cognitive functions, such as attention and working memory (Jang and Kwon, 2017; Kim et al., 2020). As the largest commissure fiber, CC is a major pathway for interhemispheric information exchange and function integration. Some studies have demonstrated that the Genu of CC contains fiber connecting the frontal poles, and the loss of these connections could disrupt working memory ability (Choudhri et al., 2013; Prunas et al., 2018; Raghavan et al., 2020). The ACR was a part of the limbic-thalamo-crotical loops and contained ascending and descending thalamic projections from the internal capsule to the cortex. The SLF is the vital association fiber connecting the frontoparietal regions. Disrupted ACR and SLF integrity has been found in multiple neuropsychiatric diseases and is correlated with working memory deficits (Davenport et al., 2010; Xiao et al., 2021). Therefore, the interrupted intra- and interhemispheric communication from disrupted white matter maybe the neural basis of motor and cognition dysfunctions in patients with chronic PI.

This study has some limitations. First, TBSS has a relatively higher probability of type I errors than the ROI-based approach; however, we adopted a more stringent statistical threshold (*P* < 0.01) to control for type I errors. Second, this cross-sectional study only revealed impaired white matter in patients with chronic PI and did not investigate longitudinal changes in white matter microstructure from the acute to the chronic period. We intend to conduct a longitudinal study to explore the dynamic changes in tissue microstructure. Third, in this study, although two non-zero *b*-values (1,000 and 2,000) and 25 diffusion gradient directions can be used to calculate diffusion and kurtosis parameters, the choice is not optimal (Chuhutin et al., 2017). A series of better DKI parameters should be performed to accurately estimate kurtosis parameters and reduce image acquisition time for further clinical applications. Finally, the relatively small sample size limits the generalizability of the findings. Future large-scale studies are needed to validate our results.

## Conclusion

In conclusion, PI could lead to extensive white matter microstructure disruption in perilesional and remote regions, and this is correlated with decreased motor and cognitive function. This maybe a vital neuronal mechanism for behavioral dysfunction in patients with chronic PI. Moreover, compared with DTI, DKI could provide more information regarding abnormal white matter microstructure, indicating that combining multiple diffusion and kurtosis parameters could contribute to a better understanding of neuropathological mechanisms in chronic PI.

## Data Availability Statement

The raw data supporting the conclusions of this article will be made available by the authors, without undue reservation.

## Ethics Statement

The studies involving human participants were reviewed and approved by the First Affiliated Hospital of Zhengzhou University and Tianjin Medical University General Hospital. The patients/participants provided their written informed consent to participate in this study. Written informed consent was obtained from the individual(s) for the publication of any potentially identifiable images or data included in this article.

## Author Contributions

YW, CW, BX, and JL conceived and designed the experiments. YW, CW, PM, LW, YYW, and SW performed MRI scans and behavioral assessments. YW, CW, and JC wrote the manuscript. SH, YRW, BX, and KW revised this manuscript. All authors contributed to the article and approved the submitted version.

## Conflict of Interest

KW was employed by company GE Healthcare and BX was employed by company Beijing Intelligent Brain Cloud, Inc. The remaining authors declare that the research was conducted in the absence of any commercial or financial relationships that could be construed as a potential conflict of interest.

## Publisher’s Note

All claims expressed in this article are solely those of the authors and do not necessarily represent those of their affiliated organizations, or those of the publisher, the editors and the reviewers. Any product that may be evaluated in this article, or claim that may be made by its manufacturer, is not guaranteed or endorsed by the publisher.

## Footnotes

1
www.nitrc.org/projects/mricron

2
http://www.nitrc.org/projects/dke

3
http://www.humanbrain.cn

## Abbreviations

MCP: Middle cerebellar peduncle
PCT: Pontine crossing tract (a part of MCP)
Genu of CC: Genu of corpus callosum
Body of CC: Body of corpus callosum
Splenium of CC: Splenium of corpus callosum
FX: Fornix (column and body of fornix)
CST: Corticospinal tract
ML: Medial lemniscus
ICP: Inferior cerebellar peduncle
SCP: Superior cerebellar peduncle
CP: Cerebral peduncle
ALIC: Anterior limb of internal capsule
PLIC: Posterior limb of internal capsule
RLIC: Retrolenticular part of internal capsule
ACR: Anterior corona radiate
SCR: Superior corona radiate
PCR: Posterior corona radiate
PTR: Posterior thalamic radiation (include optic radiation)
SS: Sagittal stratum (include inferior longitudinal fasciculus and inferior fronto_occipital fasciculus)
EC: External capsule
CG: Cingulum (cingulate gyrus)
CH: Cingulum (hippocampus)
ST: Fornix (cres)/Stria terminalis
SLF: Superior longitudinal fasciculus
SFO: Superior fronto_occipital fasciculus (could be a part of anterior internal capsule)
UNC: Uncinate fasciculus
TAP: Tapetum.
